# Magnolin alleviated DSS‐induced colitis by inhibiting ALOX5‐mediated ferroptosis

**DOI:** 10.1002/kjm2.12806

**Published:** 2024-02-10

**Authors:** Ting Yao, Yuan‐Yuan Yao, Jin‐Zhi Wang, Shi‐Man Jiang, Lan‐Juan Li

**Affiliations:** ^1^ State Key Laboratory for Diagnosis and Treatment of Infectious Diseases, National Clinical Research Center for Infectious Diseases, National Medical Center for Infectious Diseases, Collaborative Innovation Center for Diagnosis and Treatment of Infectious Diseases, The First Affiliated Hospital Zhejiang University School of Medicine Hangzhou City China

**Keywords:** ALOX5, ferroptosis, inflammatory bowel disease, macrophages, Magnolin

## Abstract

Inflammatory bowel disease (IBD) is a chronic and incurable disorder associated with higher cancer risk and currently faces unsatisfactory treatment outcomes. Ferroptotic cells secrete damage‐associated molecular patterns (DAMPs) that recruit and activate immune cells, particularly macrophages. Magnolin has excellent antioxidant and anti‐inflammatory properties, but its effect on IBD has not yet been clearly understood. This study aimed to investigate the therapeutic effects and mechanism of magnolin in IBD. For this purpose, in vivo and in vitro colitis models were established using dextran sulfate sodium (DSS), followed by optimization of magnolin concentration 2.5 μg/mL in vitro and 5 mg/kg in vivo. Bioinformatics analysis identified potential magnolin target sites and evaluated ferroptosis‐associated gene expressions. Body weight, food intake, disease activity index (DAI), pathological changes, and inflammation levels were assessed. The effect of magnolin on ferroptosis and macrophages was evaluated using quantitative real time‐polymerase chain reaction (qRT‐PCR), immunofluorescent staining, flow cytometry, enzyme‐linked immunosorbent assay (ELISA), and western blotting. Results indicated that magnolin at a lower dose (5 mg/kg) alleviated DSS‐induced colitis symptoms and reduced inflammation in mice. The bioinformatics analysis showed arachidonate 5‐lipoxygenase (ALOX5) as a potential magnolin target. Furthermore, magnolin inhibited the expression of *ALOX5* with no effect on GPX4. Moreover, magnolin regulated macrophage differentiation into the M2 phenotype and suppressed pro‐inflammatory factors, that is, interleukin‐6 and tumor necrosis factor‐α (IL‐6 and TNFα). These results suggested that magnolin possesses significant therapeutic potential in treating IBD by suppressing *ALOX5*‐mediated ferroptosis, inhibiting M1 while promoting M2 macrophages, which is envisaged to provide novel strategies for treating IBD.

## 1 INTRODUCTION

Inflammatory bowel disease (IBD) is characterized by the disruption of epithelial barrier functionality and persistent inflammation of the mucosa, comprising Crohn's disease and ulcerative colitis (UC).^1^, ^2^ Regardless of geographical variations, IBD burdens healthcare systems considerably, displaying a high and escalating prevalence globally.^3^ Although IBD exhibits a relatively low mortality rate, it is associated with significant morbidity and an unsatisfactory curative outcome and can progress into colon cancer if left untreated.^4^ Alongside genetic susceptibility, the advancement of IBD is hypothesized to be associated with an aberrant mucosal response to environmental factors and the gut microbiota; however, the precise etiology remains elusive.^5^ IBD frequently manifests as impaired intestinal barrier integrity, resulting from dysfunctional tight junctions or epithelial injury, consequently leading to the infiltration of diverse inflammatory cells, including macrophages.^6^ The primary therapeutic approaches for moderate to severe IBD involve administering immunosuppressive agents. Nevertheless, these interventions encounter challenges like drug tolerance development and significant adverse effects.^3^ Therefore, it is imperative to enhance our comprehension of IBD pathogenesis and identify novel signaling pathways to address the unmet therapeutic requirements of IBD‐affected patients.

Many studies have highlighted cell death's significance in maintaining intestinal epithelial cells (IECs) homeostasis. Ferroptosis, a regulated form of cell death, is distinguished by iron‐dependent lipid peroxidation, particularly affecting phospholipids containing polyunsaturated fatty acids (PUFAs) within cell membranes.^7^ Lipid peroxidation primarily impacts PUFAs in biological membranes, negatively affecting cellular function. Iron, apart from facilitating lipid peroxidation through non‐enzymatic mechanisms, induces ferroptosis by augmenting the activity of arachidonate lipoxygenase (ALOX).^7^ Ferroptosis necessitates the accumulation of ω‐6‐PUFA arachidonic acid (AA) within cell membranes, facilitated by acyl‐CoA synthetase long‐chain family member 4 (ACSL4), serving as a substrate for lipid peroxidation.^8^, ^9^ Nevertheless, various antioxidant systems, including the GPX4/GSH, contribute to mitigating the effects of oxidative stress. Lipid peroxidation not only impairs cell membranes but also stimulates the release of damage‐associated molecular patterns (DAMPs), including extracellular high mobility group box 1 (HMGB1) and host DNA, as well as oxidized lipid metabolites like malondialdehyde (MDA) and 4‐Hydroxynonenal (4‐HNE), provoking inflammatory and immune responses.^7^ IBD was characterized by excessive inflammation in the colon resulting from immunological imbalance, apparent observations include the accumulation of ferrous iron (Fe^2+^), generation of reactive oxygen species (ROS), and lipid peroxidation. Numerous studies have demonstrated that iron chelators can mitigate ROS production and alleviate colonic symptoms in IBD.^10^, ^11^ However, ferroptosis's specific role and mechanism in IBD necessitate further investigation.

*Magnolia*, a traditional medicinal plant, has been shown to have diverse physiological effects, including mitigation of ROS and lipid oxidation^12^ and anti‐inflammatory effects^13^ by regulating inflammatory mediators, fatty acid, and cholesterol metabolism.^14^, ^15^ Magnolin, derived from *Magnolia* flos, is shown to exhibit potent therapeutic effects in various inflammatory conditions, such as inflammatory periodontal disease,^16^ osteoarthritis,^17^ and pulmonary inflammation.^18^ Furthermore, the extracts from the *Magnolia* genus have been used in treating colitis.^19^, ^20^ Nonetheless, the potential of magnolin to alleviate IBD and its specific impact on ferroptosis remains unexplored.

This study aimed to investigate the therapeutic potential of magnolin and its mechanism in regulating ferroptosis‐associated molecules as a novel IBD treatment strategy.

## 2 MATERIALS AND METHODS

### 2.1 Chemicals and reagents

Magnolin was purchased from Yesheng (Shanghai, China, purity ≥98%, molecular weight = 416.5). Phosphate buffered saline (PBS), RPMI 1640 medium, penicillin‐streptomycin, and fetal bovine serum (FBS) were obtained from Gibco (Grand Island, NY, USA). The following antibodies were purchased from BioLegend (San Diego, CA, USA): anti‐PerCP‐Cy5.5 CD11b, anti‐FITC‐F4/80, anti‐PE‐CD86, and anti‐APC‐CD206, while anti‐ALOX5, GPX4, ACSL4, and GAPDH were obtained from Proteintech (Wuhan, China). Ferrostatin‐1 (Fer‐1) and RSL3 were procured from MCE (Shanghai, China); DSS and aminosalicylate (5‐ASA) were purchased from Macklin (Shanghai, China). BODIPY 581/591 C11 and CCK8 kit were acquired from GLPBIO (California, USA). Animal total RNA isolation kit, ABScript III RT Master Mix, and SYBR Green reagents were obtained from ABclonal Technology (Shanghai, China). MDA assay kit was purchased from Beyotime (Shanghai, China); the Fe^2+^ content assay kit from Solarbio (Beijing, China), and FITC‐dextran (average molecular weight 3000 to 5000 Da) was purchased from Sigma–Aldrich (Steinheim, Germany).

### 2.2 Animals models

Male C57BL/6 mice (age = 6 weeks) were procured from Ziyuan company (Hangzhou, Zhejiang, China) and housed in a specific pathogen‐free (SPF) environment with a 12 h light–dark cycle. Following a 1‐week acclimatization, animals underwent a modeling procedure using established methods, while 5‐ASA, a commonly used drug for treating IBD, served as a positive control. All animals were randomly divided into five groups with *n* = 6/group, and labeled as NC (PBS and no DSS), DSS (PBS and 2.5% DSS), the DSS + magnolin 5 mg/kg (5 mg/kg body weight/day of magnolin + 2.5% DSS), DSS + magnolin 10 mg/kg group (10 mg/kg body weight/day of magnolin + 2.5% DSS), and DSS + 5‐ASA group (150 mg/kg body weight/day of 5‐ASA + 2.5% DSS). All animals received their respective treatments orally from day one of the DSS intervention, which continued for 7 days. Animals were anesthetized and euthanized, and relevant samples were collected on the 8th day and stored at −80°C until further use.

For assessment of intestinal permeability, following overnight fasting, 4 h before blood collection, mice were administered FITC‐dextran (50 mg/100 g body weight) orally, and fluorescence intensities were quantified at 490/525 nm. The animal protocol was approved by the Animal Care and Use Committee of the First Affiliated Hospital, School of Medicine, Zhejiang University (20231329).

### 2.3 Bioinformatics analysis

The GSE66407 dataset was obtained from GEO and analyzed with R software (version 4.1.3). After pre‐processing the raw data, obtaining log2 expression values, and normalizing them with the “affy” R package, the “limma” package was employed to identify differentially expressed genes (DEGs). The expression levels of the relevant genes were visualized with the “ggplot2” package, and the network tool CIBERSORT was used to analyze immune infiltration.

### 2.4 Homology modeling

The 3D structure of ALOX5 (PDB ID: 7TTK) was retrieved from the Protein Data Bank. The ligand molecules preparation and their interaction with protein receptors were conducted using AutoDockTools (version 1.5.6). For this purpose, the AutoGrid parameter file was utilized for grid box representation, and subsequently, the AutoDock parameter file was configured and executed. The candidate ligand molecules were evaluated based on their docking interactions with the target protein, and the ones showing the most favorable interactions were selected for further analysis. The visualization of the results was conducted using PyMOL software. To validate the potential binding affinity between the core target and the active compound, a docking score of ≥4.25 was applied.

### 2.5 Cell culture and treatment

NCM460 and THP‐1 cells were obtained from Saibaikang Biological Company (Shanghai, China) and cultured in RPMI‐1640 medium supplemented with 10% FBS, penicillin (100 IU/mL), and streptomycin (100 μg/mL). In vitro colitis models were established using a previously described protocol.^21^ Briefly, NCM460 cells were subjected to an overnight serum‐free starvation, followed by a 4 h treatment with 2% DSS. Subsequently, these cells were incubated with different concentrations of magnolin (0, 2.4, 6, 12, and 24 μM) or Fer‐1 (4 μM) in a reduced serum culture medium. In subsequent in vitro cell experiments, a 6 μM magnolin concentration was used and the 3 groups were set up: Normal group (no DSS treatment + PBS), Control group (2% DSS + PBS) and Magnolin group (2% DSS + 6 μM magnolin). Ferroptosis was induced in cells by incubating them with RSL3 (5 μM) and magnolin for 24 h.

To explore the relationship between IECs and macrophages, THP‐1‐derived macrophages (TDMs) were generated by treating THP‐1 monocytes with 100 ng/mL phorbol 12‐myristate 13‐acetate (PMA) for 24 h. The culture medium was then replaced with supernatant obtained from NCM460 cells treated with RSL3 for 24 h, with or without magnolin.

### 2.6 Cell viability assay

Cell viability was assessed using a CCK‐8 at a wavelength of 450 nm, as per kit manufacturer's guidelines. Briefly, NCM460 cells were seeded in 96‐well plates and exposed to different treatments, followed by the replacement of the medium with CCK‐8 reagent, and incubated for 2 h, followed by detecting cell viability.

### 2.7 MDA and Fe^2+^ content assays

The MDA and Fe^2+^ concentrations in tissues or cells were quantified using the lipid peroxidation MDA assay kit and the Fe^2+^ content assay kit, respectively. Both experiments were conducted per the manufacturer's instructions provided with the kit.

### 2.8 Extraction of splenic macrophages

Spleens were harvested from animals after euthanasia. The spleens were cut into pieces and homogenized using a 5 mL syringe in ice‐cold PBS. Red blood cells were removed using red blood cell lysis buffer, and the remaining cells were washed twice with PBS and resuspended in a complete medium for 6 h. Unadherent cells were subsequently separated from the suspension.

### 2.9 Flow cytometry

For lipid peroxidation detection, the NCM460 cells were incubated with BODIPY 581/591 C11 (5 μM) at room temperature under dark for 30 min and measured at 488/560 nm. For macrophage phenotype identification, the cells were treated with PerCP‐Cy5.5 CD11b, FITC‐F4/80, PE‐CD86, and APC‐CD206 antibodies at room temperature under dark for 15 min. The cells were then gently washed with PBS and subjected to flow cytometry analysis (Beckman CytoFlex).

### 2.10 Transmission electron microscopy (TEM)

Colonic tissues were fixed with 2.5% glutaraldehyde and stained with saturated uranium acetate and lead citrate. Respective sections were photographed using TEM (HT7700, Japan).

### 2.11 Detection of gene expression level

To assess gene transcription levels, total mRNA was extracted from cells or colon tissue using the animal total RNA isolation kit and subjected to reverse transcription with ABScript III RT Master Mix. The mRNA levels were quantified using SYBR Green assay with commercial reagents employing the comparative cycle method (2^−ΔΔCT^), standardized using GAPDH as the reference. The primers employed for amplifying the target genes are provided in Table S1.

Protein levels were detected using western blotting. Briefly, proteins were extracted from lysed cells and subjected to concentration determination, electrophoresis, membrane transfer, and subsequent incubation with anti‐*GPX4*, ‐*ACSL4*, and ‐*ALOX5* primary antibodies over night at dilution of 1:1000, followed by incubation with secondary antibodies for 2 h. GAPDH served as an endogenous control. The protein bands' density was measured and assessed using ImageJ software.

The levels of interleukin‐6 (IL‐6), tumor necrosis factor‐α (TNFα), and interleukin‐10 (IL‐10) in serum samples were determined using ELISA kits (Mlbio, Shanghai, China) as per manufacturer's instructions.

### 2.12 Histochemical and immunohistochemical staining

Following immediate collection, colon tissues were washed with PBS and fixed with 4% paraformaldehyde. Subsequently, the tissues underwent paraffin embedding, sectioning into 5‐μm‐thick slices, and dewaxing treatment. Histopathological examination and morphometric analysis were conducted by staining the tissues with hematoxylin and eosin (H&E), and the disease severity scores were assessed as described in a previous study.^22^ Furthermore, the sections were subjected to immunohistochemical examination using corresponding antibodies to detect GPX4, ACSL4, and ALOX5 content and distribution.

### 2.13 Statistical analysis

Statistical data was analyzed employing the GraphPad Prism (version 8) and presented as the mean ± SD unless specified mentioned. The statistical significance of the data was analyzed using two‐way ANOVA and the S‐N‐K method for multiple group comparisons when data was normally distributed. For data with a non‐normal distribution, Kruskal–Wallis tests were performed. *p*‐values <0.05 were regarded as statistically significant.

## 3 RESULTS

### 3.1 Evaluation and determination of the protective concentration of magnolin

**FIGURE 1:**
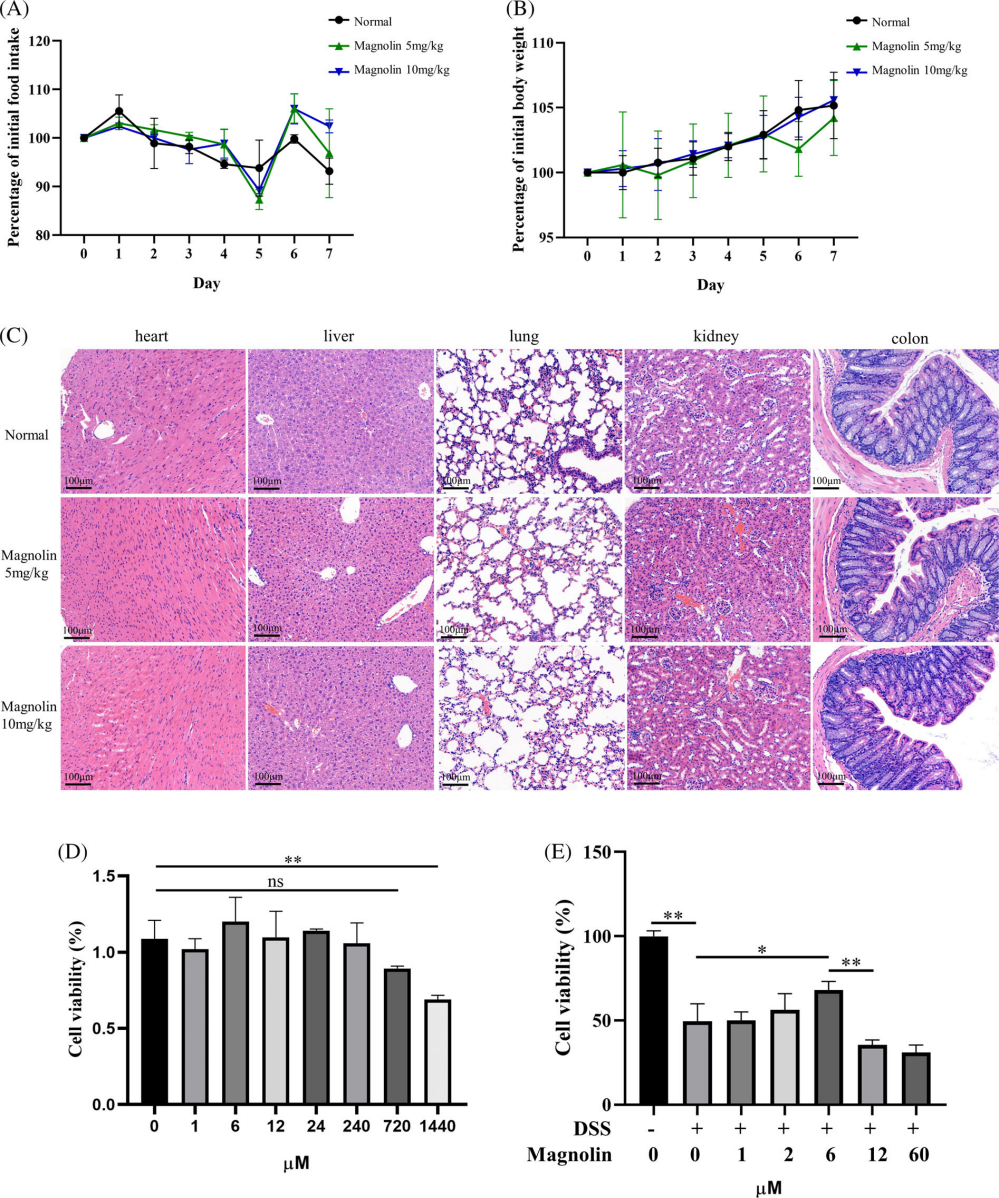
Toxicity of magnolin in NCM460 cells and normal mice. (A) Effect on food intake in normal mice (*n* = 5). (B) Effect on body weight in normal mice (*n* = 5). (C) Effect on organs in normal mice (*n* = 5, scale bar: 100 μm). (D) Cell viability at different concentrations of magnolin in normal NCM460 cells. (E) Cell viability at different concentrations of magnolin in an in vitro colitis model using NCM460 cells (*p < 0.05; **p < 0.01).

To determine an effective and safe concentration of magnolin, we conducted cytotoxicity assessments on both cell lines and mice. The findings indicated an insignificant effect on parameters such as body weight (Figure 1A), food intake (Figure 1B), and the morphology of vital organs (Figure 1C). The in vitro toxicity results indicated a substantial safety window of magnolin, with insignificant toxicity observed until concentration reaching 720 μM in normal NCM460 cells (Figure 1D). As established in a previous study,^21^ we aimed to identify a protective concentration of magnolin for in vitro colitis model. Cell viability significantly decreased at a 2% DSS concentration, while there was a steep decline in cell viability at a 3% concentration (Figure S1A); hence, a 2% DSS concentration was selected for subsequent experiments. Nevertheless, NCM460 cells exhibited diminished tolerance to magnolin, as shown by its ability to effectively mitigate DSS‐induced damage at a concentration of 6 μM, whereas higher magnolin concentrations were observed to hinder cell viability (Figure 1E).

### 3.2 Magnolin ameliorated symptoms of DSS‐induced colitis

**FIGURE 2:**
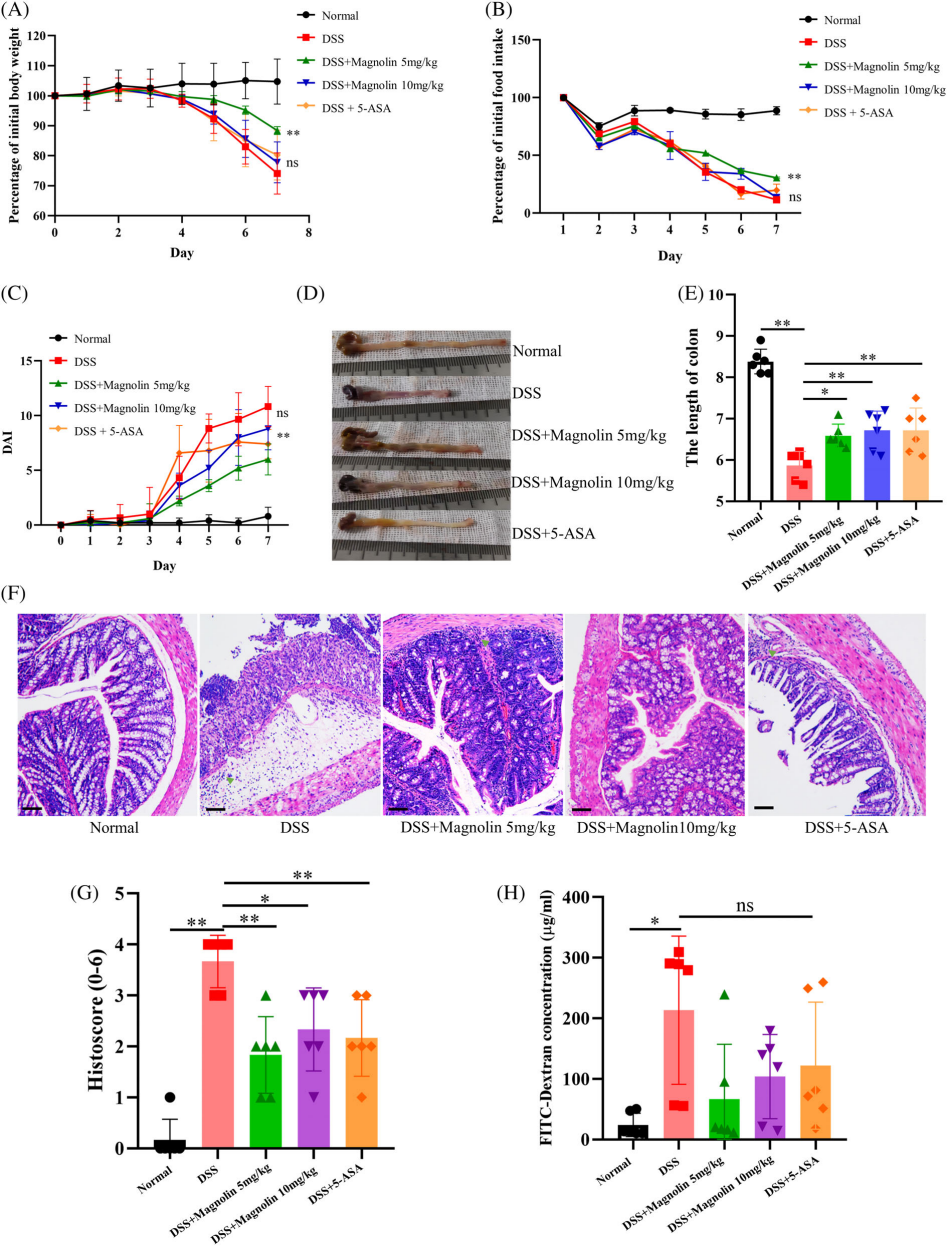
Magnolin alleviated DSS‐induced colitis in mice. (A) Effects on body weight (*n* = 6). (B) Effects on food intake (*n* = 6). (C) Effects on DAI (*n* = 6). (D, E) Effects on colon length (*n* = 6). (F) Representative pathological images of each group's colon (scale bar: 50 μm), green arrow: immune cells. (G) Histoscore of each group's colon (*n* = 6). (H) Intestinal permeability testing using FITC‐dextran (*n* = 6) (*p < 0.05; **p < 0.01). DSS, dextran sulfate sodium.

During 7 days of DSS exposure, the weight of mice in the DSS group exhibited a significant decrease, starting from the 4th day, compared to the NC group. However, the administration of a low dose of magnolin (5 mg/kg) mitigated the weight loss, whereas the high dose (10 mg/kg) failed to show an evident protective effect (Figure 2A). Similar trends were observed for the reduction in food intake (Figure 2B) and the DAI score (Figure 2C). Notably, the group treated with magnolin demonstrated similar protective effects on colon length (Figure 2D,E) and pathological injury (Figure 2F,G) at both high and low doses. Moreover, the magnolin‐treated group exhibited a restoration of colonic epithelial structure and a reduction in immune cell infiltration. Since IECs damage led to an escalation in intestinal permeability, facilitating bacterial translocation and exacerbating inflammation, intestinal permeability was assessed, and results revealed that magnolin alleviated the DSS‐induced increase in intestinal permeability (Figure 2H).

### 3.3 Magnolin ameliorated inflammation in experimental colitis

**FIGURE 3:**
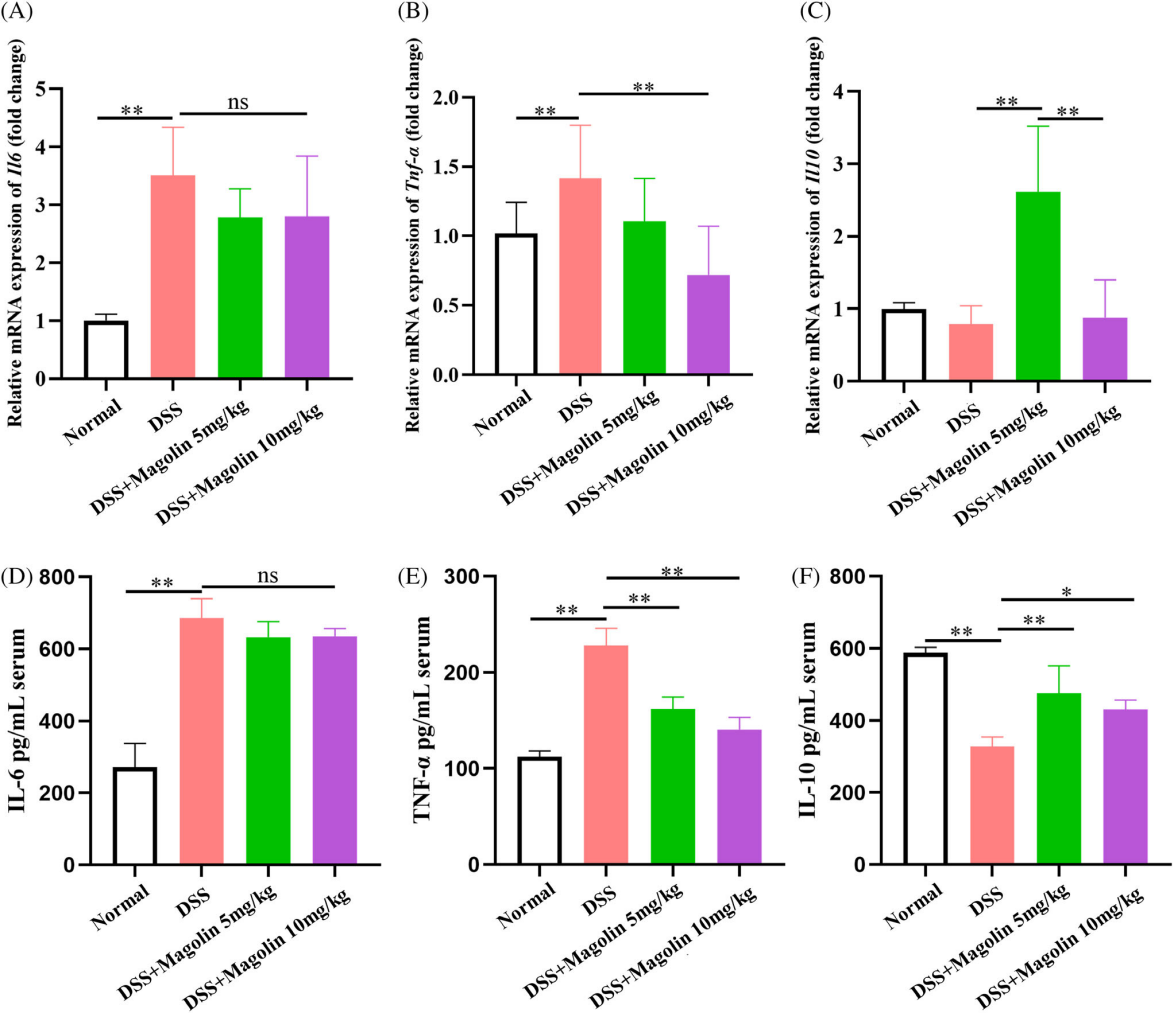
Magnolin reduced colonic inflammation in mice. (A–C) mRNA levels of IL‐6, TNFα, and IL‐10 in the colon. (D–F) Levels of the inflammatory factors IL‐6, TNFα, and IL‐10 in the serum (*p < 0.05; **p < 0.01).

The qRT‐PCR results demonstrated significantly elevated levels of pro‐inflammatory factors (IL‐6 and TNFα) in colons of the colitis animal model, while no significant change in the levels of anti‐inflammatory cytokines (IL‐10) was observed (Figure 3A–C). Administering magnolin at 10 mg/kg dose not only reduced the levels of TNFα but also of IL‐10 compared to 5 mg/kg, while the difference in the levels of IL‐6 was statistically insignificant. Subsequently, the inflammatory factors levels in serum samples were also assessed, and results were consistent with qRT‐PCR findings. The magnolin treatment induced a reduction in TNFα levels in a dose‐dependent manner, but no noticeable change was observed in IL‐6 levels, while levels of IL‐10 were higher in the 5 mg/kg compared to the 10 mg/kg group (Figure 3D–F). These results suggested that magnolin could suppress the pro‐inflammatory cell activation while higher doses might harm anti‐inflammatory cells (*p < 0.05; **p < 0.01).

### 3.4 ALOX5 as a promising target of magnolin

**FIGURE 4:**
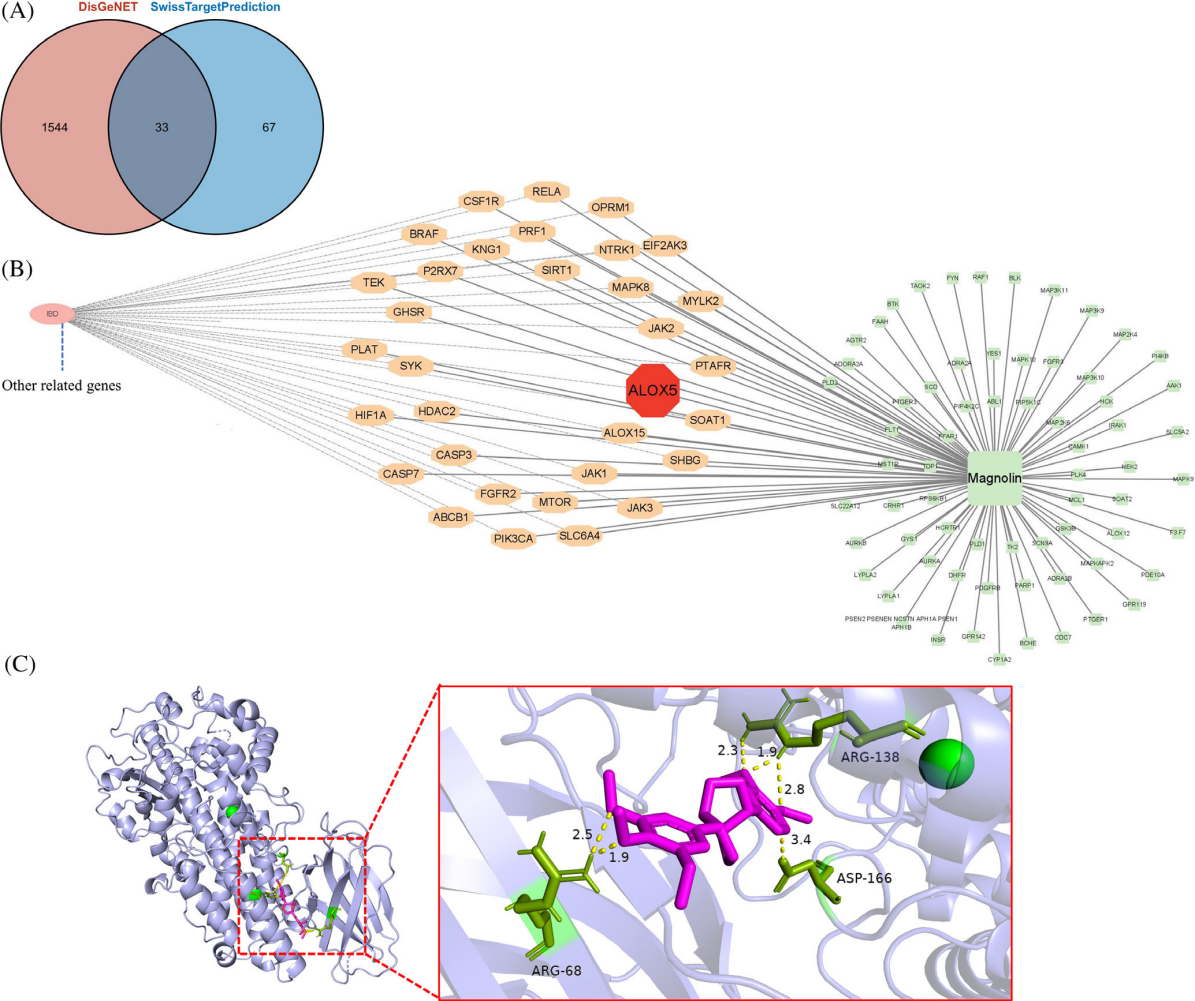
ALOX5 as a potential target of magnolin. (A, B) Common genes associated with colitis and predicted binding sites of magnolin. (C) Magnolin forms six polar bonds with ALOX5.

In our research, we utilized the Swiss target prediction web tool to identify potential therapeutic targets and understand the biological effects of magnolin. Furthermore, we incorporated data from the DisGeNET database containing IBD‐associated genes, which resulted in the discovery of 33 potential targets (Figure 4A,B), among which genes linked to ferroptosis like *MCL1*, *ALOX5*, and *ALOX15* were observed. Considering the documented upregulation of ferroptosis in patients with IBD and the significant prediction of *ALOX5* as a magnolin active site, we postulated that the therapeutic effects of magnolin might be attributed to its modulation of the ferroptosis through interaction with *ALOX5*. To investigate this further, we conducted molecular docking studies, employing AutoDockTools to examine the binding between magnolin and human *ALOX5*, and results indicated a favorable binding energy of −5.42 kcal/mol, suggesting a strong interaction between magnolin and *ALOX5*. Specifically, magnolin formed six polar bonds with human *ALOX5*, facilitating complex stabilization and potentially influencing the structure or function of *ALOX5*. These bonds were explicitly associated with the amino acids ARG68, forming two polar bonds at distances of 2.5 Å and 1.9 Å; ARG138, forming bonds at 2.3 Å, 1.9 Å, and 2.8 Å, and ASP166, forming a bond at 3.4 Å within the *ALOX5* protein (Figure 4C). These findings provided compelling evidence supporting the plausibility of the direct binding interaction between magnolin and *ALOX5*.

### 3.5 Magnolin suppressed ferroptosis in NCM460 cells

**FIGURE 5:**
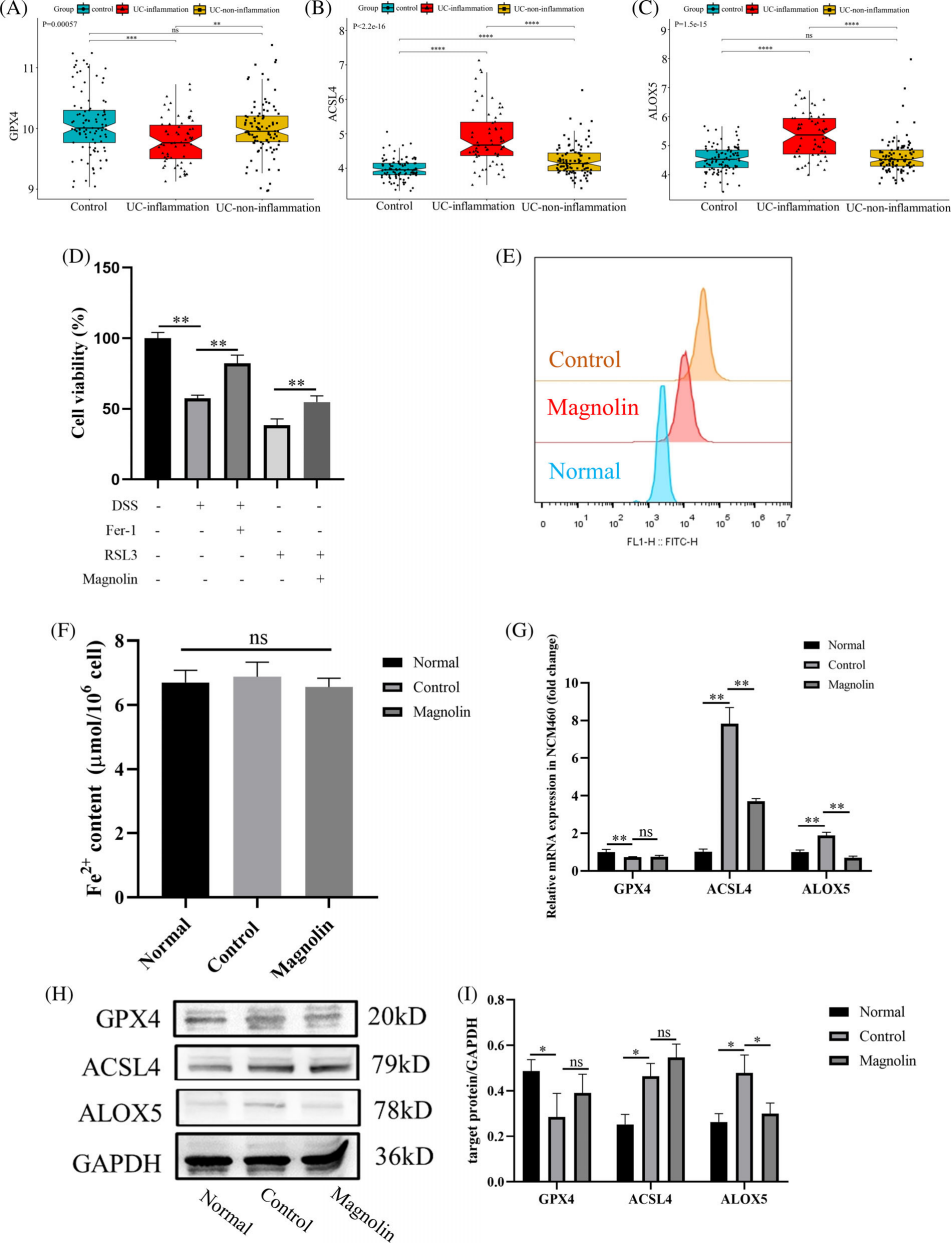
Magnolin reducer ferroptosis in NCM460 cells. (A–C) Expression of *GPX4*, *ACSL4*, and *ALOX5* in UC patients. (D) Cell viability under different DSS, Fer‐1, RSL3, and magnolin treatments. (E) Lipid peroxidation level was assessed using BODIPY581/591 C11. (F) Fe^2+^ content in cells. (G) mRNA expression of *GPX4*, *ACSL4*, and *ALOX5* in NCM460 cells. (H, I) Protein expression of *GPX4*, *ACSL4*, and *ALOX5* in NCM460 cells (*p < 0.05; **p < 0.01).

To investigate whether magnolin alleviates IBD by inhibiting ferroptosis, we first utilized the pre‐existing database GSE66407 from GEO, comprising 97 normal, 62 UC in an inflammatory state, and 99 non‐inflammatory state samples. As depicted in Figure S2 and Figure 5A–C, the expression of *GPX4*, a crucial enzyme in the GSH system responsible for combating oxidative stress, was reduced, while *ACSL4*, a critical molecule in fatty acid metabolism, and *ALOX5*, a promoter of ferroptosis, were increased. Apart from the GPX4/GSH system, GCH1, DHODH, and FSP1 (AIFM2) represented other antioxidant systems. Nonetheless, the analysis of the first two molecules' expression revealed insignificant statistical differences, while FSP1 was downregulated in the inflammation group as opposed to the control group. Furthermore, PTGS2 was upregulated, while FTL and FTH1 were downregulated. In vitro experiments, Fer‐1, a ferroptosis inhibitor, mitigated DSS‐induced cell damage (Figure 5D). These findings proved that ferroptosis occurs in UC patients. Subsequently, we observed that magnolin (2.5 μg/mL) reduced cellular injury induced by RSL3, a synthetic compound that induces ferroptosis by inactivating GPX4^23^ (Figure 5D), indicating that magnolin effectively reduced ferroptosis. It also reduced DSS‐induced lipid peroxidation in NCM460 cells (Figure 5E) based on C11‐BODIPY staining. However, the concentration of Fe^2+^ in cells did not exhibit significant changes, suggesting that the occurrence of ferroptosis may not be directly related to iron metabolism (Figure 5F). In further experiments, we measured the levels of *GPX4*, *ACSL4*, and *ALOX5* in cells at the transcription and translation levels. Consistent with the patient's data, DSS‐induced cell damage led to a decrease in *GPX4* and an increase in *ACSL4* and *ALOX5* levels. Magnolin was found to downregulate *ALOX5* but showed no significant effect on *GPX4* and *ACSL4* (Figure 5G–I). These results suggested that magnolin suppressed ferroptosis by targeting *ALOX5* in vitro model.

### 3.6 Magnolin suppressed ferroptosis in IECs in mice with colitis

**FIGURE 6:**
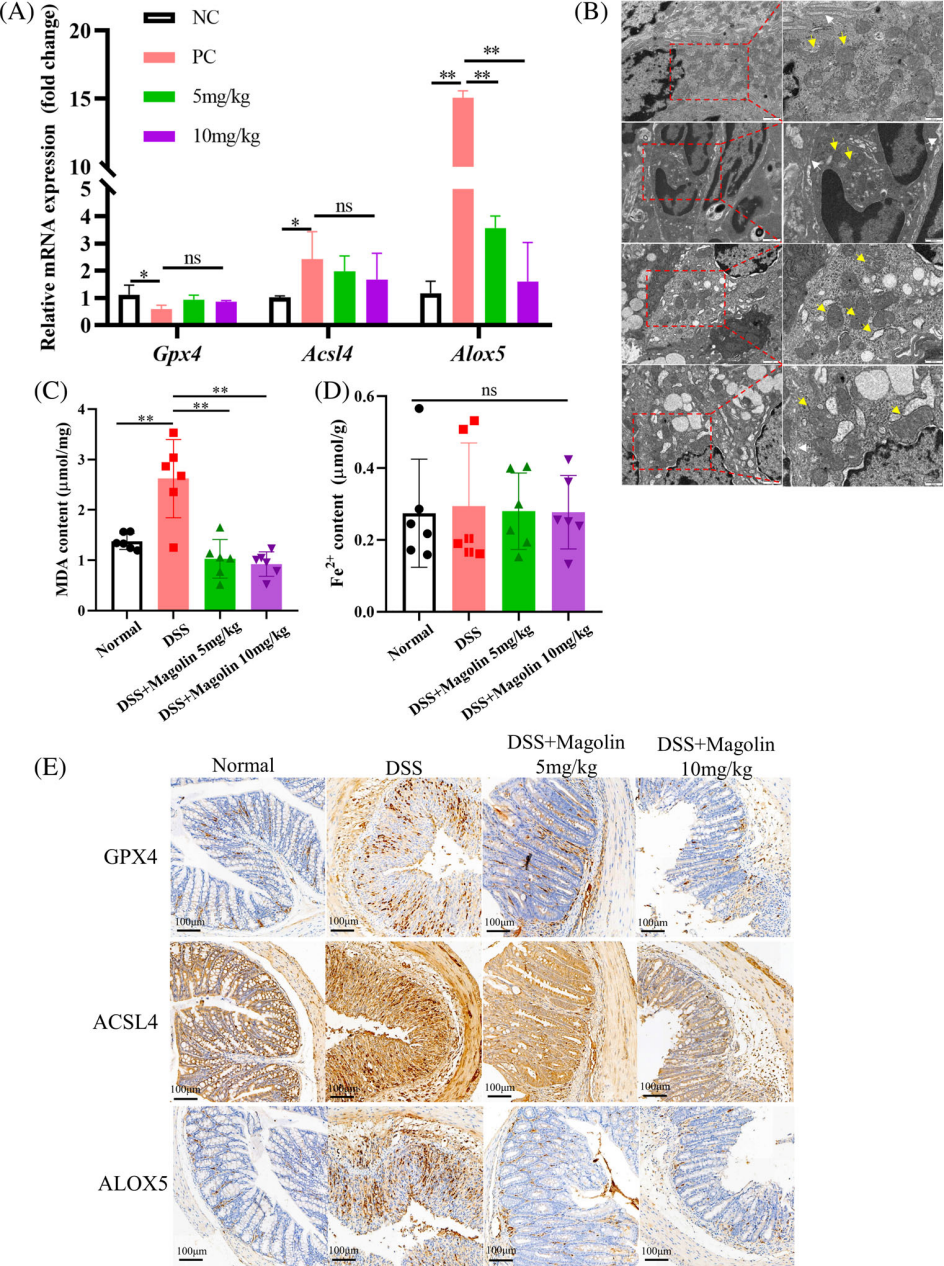
Magnolin reduced ferroptosis in vivo. (A) mRNA expression of *GPX4*, *ACSL4*, and *ALOX5* in colon tissue. (B) Representative images of mitochondria in each group's colon (yellow arrow) and tight junctions between IECs (white arrow) (scale bar: 500 nm). (C) MDA content in colon tissue. (D) Fe^2+^ content in colon tissue. (E) Representative images of protein expression levels of *GPX4*, *ACSL4*, and *ALOX5* (scale bar: 100 μm) (*p < 0.05; **p < 0.01).

Consistent with the results from in vitro experiments, we observed a reduction in the expression of *Gpx4*, while *Acsl4* and *Alox5* were upregulated in the DSS‐induced colitis model at the transcription level (Figure 6A). Following the administration of magnolin, the expression of *Alox5* significantly decreased, and *Acsl4* showed a downward trend while there was no observed change in *Gpx4* expression. Subsequently, we employed a TEM to examine the morphology of IECs. Within the DSS group, we observed mitochondria with reduced size, increased membrane density, decreased or disappeared mitochondrial cristae, and ruptured outer mitochondrial membrane. However, in the magnolin group, the mitochondrial morphology appeared relatively normal (Figure 6B). Magnolin effectively reduced the levels of MDA induced by DSS (Figure 6C) but yielded no statistically significant variation in the concentration of Fe^2+^ across the groups (Figure 6D). The changes at translation level were consistent with transcription level; however, *GPX4* was higher in the DSS group than in NC group, which might be a response to oxidative stress (Figure 6E).

### 3.7 Magnolin regulated the polarization of macrophages

**FIGURE 7:**
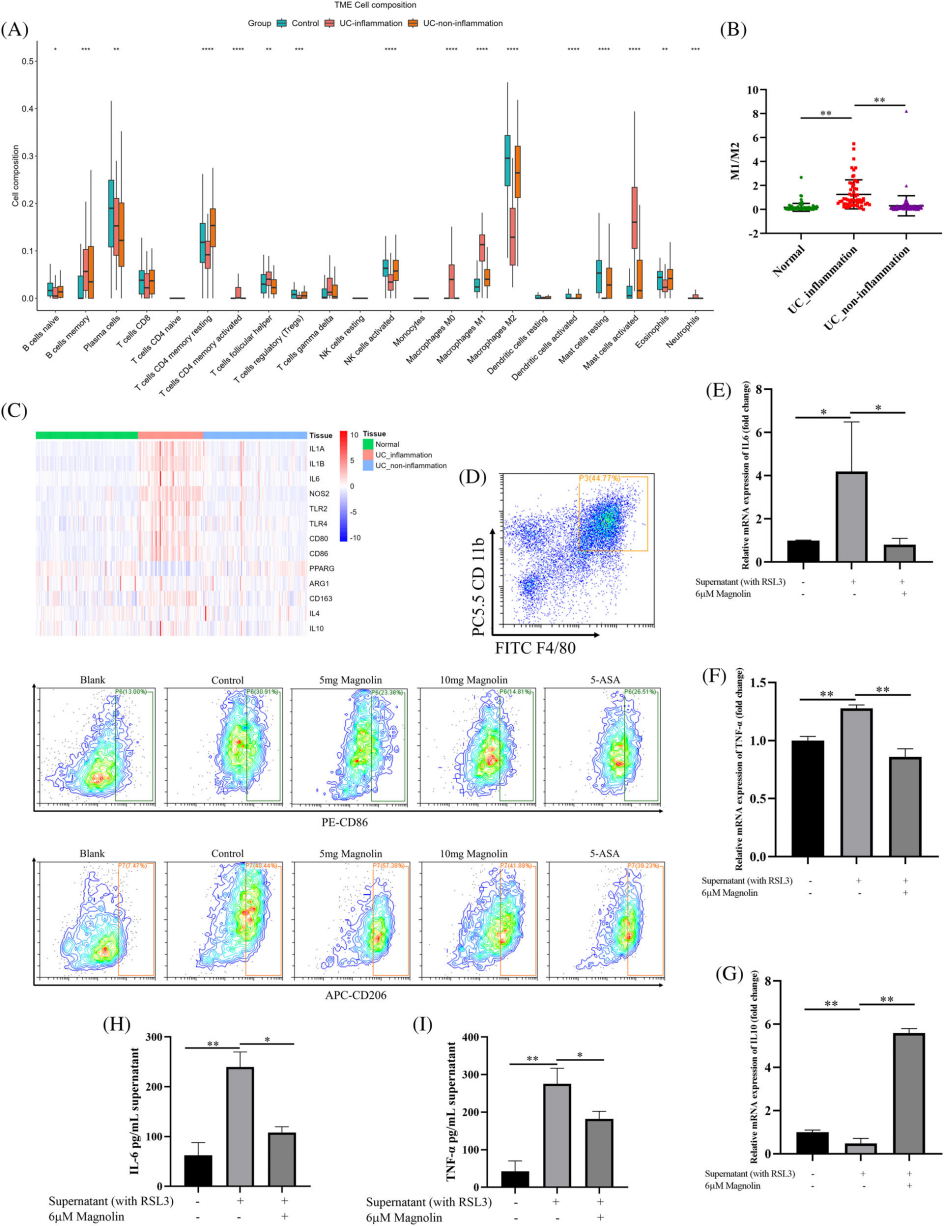
Magnolin modulated macrophage polarization. (A) Levels of various immune cells in UC patients. (B) The M1/M2 ratio in UC patients. (C) mRNA expression of related M1 and M2 marker genes. (D) M1 and M2 levels in spleen‐derived macrophages detected by flow cytometry. (E–G) mRNA expression of IL‐6, TNFα, and IL‐10 in THP‐1‐derived macrophages. (H, I) Cytokine of IL‐6 and TNFα in the supernatant of THP‐1‐derived macrophages (*p < 0.05; **p < 0.01).

Given the pivotal role of immunity in the pathogenesis of IBD, our investigation entailed an in‐depth analysis of immune cell infiltration in patients in GSE66407 database. In particular, our observations revealed elevated levels of M1 and concomitantly reduced levels of M2 macrophages (Figure 7A). Moreover, the ratio of M1/M2 and the expression of molecules associated with M1 were found to be elevated in patients with inflammation (Figure 7B,C). Subsequently, we isolated splenic macrophages from mice to investigate their function. Our results demonstrated that administering magnolin at 5 mg/kg reduced the number of M1 cells and increased the number of M2 cells. Nonetheless, 10 mg/kg magnolin significantly enhanced the inhibitory effect on M1 and M2 cells. To elucidate whether the effect of magnolin on macrophages was attributed to the reduction of ferroptosis in IECs or its direct influence on macrophages, we conducted an experiment involving the addition of the supernatant from NCM460 cells treated with RSL3 to TDMs. Our findings indicated that the supernatant stimulation prompted macrophages to differentiate toward the M1 phenotype. However, when magnolin was added, it reduced the expression of pro‐inflammatory factors (IL‐6 and TNFα) while enhancing the secretion of the anti‐inflammatory factor (IL‐10) (Figure 7D–F). In conclusion, regulating macrophages by magnolin directly affected macrophages and reduced ferroptosis of IECs.

## 4 DISCUSSION

IBD remains without a definitive cure and is associated with a high cancer risk. The demise of IECs and the presence of heightened inflammation had been regarded as significant contributors to the onset and severity of IBD. In our current study, we successfully identified the occurrence of ferroptosis in UC patients and the DSS‐induced colitis model. Our findings demonstrated that magnolin effectively ameliorated colitis by mitigating *ALOX5*‐mediated ferroptosis in IECs and concurrently suppressing pro‐inflammatory cytokine production.

Magnolin, a vital regulator of cell proliferation and inflammation, exerted its effects by inhibiting the extracellular signal‐regulated kinase pathway.^24^, ^25^ Additionally, other studies indicated magnolin targeted NF‐κB in LPS‐stimulated RAW264.7 cells.^16^ In our research, we demonstrated the effectiveness of magnolin in alleviating symptoms and inflammation in DSS‐induced colitis. However, intriguingly, we observed that the therapeutic effect at 10 mg/kg was not superior to the 5 mg/kg dose, particularly regarding weight and food intake. We proposed a hypothesis that magnolin at a high dose might inhibit both ferroptosis and the regeneration of IECs. Numerous studies showed that magnolin inhibited cell proliferation in different tissues at different concentrations, such as lung cancer,^26^ ovarian cancer cells,^24^ hepatocellular carcinoma,^25^ and prostate cancer.^27^ However, it is worth noting that the dosage required for cancer treatment is typically higher than that used for managing inflammatory conditions, such as allergy^28^ and osteoarthritis.^17^ Another possibility is that at high concentrations, drugs are not fully absorbed and utilized because the absorptivity of the drug is influenced by blood flow, which is reduced in colitis.^29^ Furthermore, our observations revealed that the higher concentration of magnolin reduced M2 macrophages, responsible for secreting anti‐inflammatory cytokines like IL‐10, which play a crucial role in tissue remodeling, wound healing, and immune responses.^30^ Since we did not observe any side effects in the colon at 10 mg/kg magnolin dose in healthy mice, we speculated that the tolerance of the damaged gut to magnolin might decrease. Nonetheless, it was important to highlight that 10 mg/kg magnolin exhibited a therapeutic effect on colitis, even though it did not surpass the effectiveness of the 5 mg/kg dose group. However, we must admit that the therapeutic window of magnolin is narrow. Triptolide, a traditional Chinese medicine with anti‐inflammatory, anti‐tumor, and anti‐autoimmune properties, was cautiously used in clinical practice due to its limited treatment window. Ongoing research is focused on combining it with western medicine^31^ or designing targeted drug delivery systems based on nanomaterials. These systems aim to decrease adverse effects and enhance efficacy by delivering drugs to specific sites of action, thereby reducing the amount of free drugs in other tissues and organs.^32^ Modifying or improving the medication method of magnolin could potentially enhance the drug's efficiency and safety. Moreover, during the examination of the inhibitory effects of different doses on mRNA and protein levels in colon tissues, we found that the 5 mg/kg magnolin effectively inhibited the upregulation of gene expression related to ferroptosis in DSS‐induced colitis. Due to the complex body environment, the mRNA and protein level changes were inconsistent. In conclusion, based on our findings, 5 mg/kg magnolin emerged as a highly effective treatment for colitis.

Ferroptosis is a complex process characterized by an intricate interplay of iron imbalance, fatty acid metabolism, and a compromised antioxidant system, however mysteries and controversies still remain.^8^, ^33^ For example, ACSL4 was found to be unnecessary for p53‐dependent ferroptosis mediated by ALOX12.^34^ Our results showed no change in the iron concentration, different from the canonical ferroptosis, which might be attributed to intracellular iron redistribution rather than the absorption of extracellular iron interdependent with lipid peroxidation.^35^ Bioinformatics analysis indicated GPX4/GSH and FSP1/CQ10 systems were disrupted in patients, which reduced lipid peroxidation by eliminating ROS. Moreover, we found that the expression of *ALOX5* was upregulated in patients and experimental colitis models. As an important member of the *ALOX* family, *ALOX5* mediated lipid peroxidation to oxidize PUFAs in cell membranes. Even worse, the oxidation products induced inflammation and triggered the formation of adducts and crosslinks in proteins or DNA, progressively impairing their function.^9^ Besides, the upregulated expression of *ALOX5* led to the production of leukotrienes, which had been implicated in the pathogenesis of IBD.^36^ Our study results revealed a significant interaction between magnolin and *ALOX5*, reducing ALOX5 expression and highlighting its potential therapeutic effect in regulating ferroptosis.

Interestingly, we noticed that the marker genes of ferroptosis returned to their normal levels when inflammation subsided, revealing a significant correlation between inflammation and ferroptosis (Figure 5A–C). Inflammation fostered the process of ferroptosis, releasing DAMPs from necrotic IECs, thereby exacerbating the inflammatory response. Multiple studies have shown that inhibiting *ALOX5* effectively alleviated inflammation.^37^, ^38^ Conversely, it has been observed that immunity could also promote ferroptosis.^39^ Our investigations further revealed ACSL4 being another ferroptosis‐related molecule influenced by magnolin.

Macrophages are closely related to disease prognosis and severity. They play a dual role, that is, releasing pro‐inflammatory cytokines and chemokines to facilitate immune cell infiltration and enhance the immune response. Simultaneously, they efficiently clear necrotic cell debris and promote cell proliferation.^30^ Furthermore, it was important to highlight that macrophages, being iron‐rich cells, are susceptible to ferroptotic damage,^7^ and the sensitivity of M1 and M2 cells to ferroptosis differed.^40^ We observed that M1 was inhibited with increasing concentrations of magnolin, while M2 decreased at high concentrations. Further studies are needed to prove whether macrophages are involved in ferroptosis and to explore the response of different subtypes of macrophages to magnolin.

**FIGURE 8:**
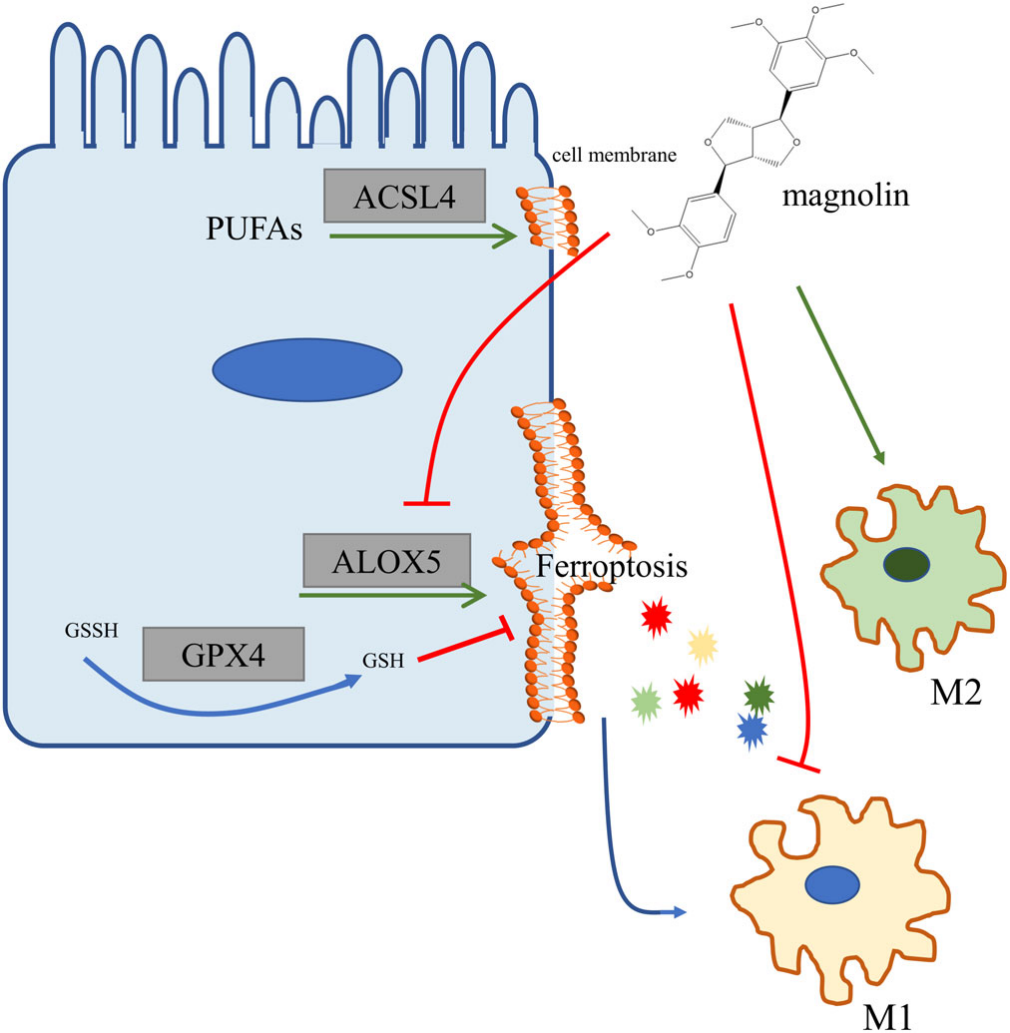
Mechanism of magnolin's ferroptosis inhibition. Magnolin suppressed the function and expression of *ALOX5*, leading to decreased ferroptosis in IECs. This reduced the release of damage‐inducing molecules, triggering macrophage differentiation into the M1 phenotype. Additionally, magnolin directly inhibited macrophage differentiation into the M1 phenotype while promoting its differentiation toward the M2 phenotype.

In conclusion, our study demonstrated that a non‐cytotoxic dose of magnolin effectively alleviated DSS‐induced colitis attributed to its inhibition of ferroptosis‐related *ALOX5*, inflammation reduction, and regulation of macrophage polarization (Figure 8). Nevertheless, further investigation is necessary to comprehend the underlying mechanism, including providing more robust evidence of the specific magnolin sites, considering its potential multiple targets. Additionally, it is imperative to elucidate the reasons behind the ineffectiveness of magnolin at high doses. However, these findings emphasized that magnolin could be a novel molecule for treating colitis.

## CONFLICT OF INTEREST STATEMENT

The authors declared no potential conflicts of interest with respect to the research, authorship, and/or publication of this article.

## Supporting information


**Data S1.** Supporting Information
