# Supplementary material for: Magnolin alleviated DSS‐induced colitis by inhibiting ALOX5‐mediated ferroptosis
Source: Kaohsiung J Med Sci. 2024 Feb 10;40(4):360–73. doi: 10.1002/kjm2.12806 (PMC11895611; doi:10.1002/kjm2.12806)
Supplement: Supplementary file 1 — Data S1. Supporting Information [file KJM2-40-360-s001.docx]

Table S1. Oligonucleotide sequences used for Q-PCR analysis.

| Species | Primer | Sequences (5'‑3') |
| --- | --- | --- |
| Human | TNFα | F 5’- GAGGCCAAGCCCTGGTATG -3’  R 5’- CGGGCCGATTGATCTCAGC-3’ |
|  | GPX4 | F 5’- GAGGCAAGACCGAAGTAAACTAC -3’  R 5’- CCGAACTGGTTACACGGGAA -3’ |
|  | IL10 | F 5’- GACTTTAAGGGTTACCTGGGTTG -3’  R 5’- TCACATGCGCCTTGATGTCTG -3’ |
|  | ACSL4 | F 5’- CATCCCTGGAGCAGATACTCT -3’  R 5’- TCACTTAGGATTTCCCTGGTCC -3 |
|  | IL6 | F 5’- CCTGAACCTTCCAAAGATGGC -3’  R 5’- TTCACCAGGCAAGTCTCCTCA -3’ |
|  | GAPDH | R 5’-CTGGGCTACACTGAGCACC-3’  F 5’-AAGTGGTCGTTGAGGGCAATG-3’ |
|  | ALOX5 | F 5’- ACAAGCCCTTCTACAACGACT -3’  R 5’- AGCTGGATCTCGCCCAGTT -3’ |
| Mouse | Tnfα | F 5’- CAGGCGGTGCCTATGTCTC -3’  R 5’- CGATCACCCCGAAGTTCAGTAG -3’ |
|  | Il10 | F 5’- CTTACTGACTGGCATGAGGATCA -3’  R 5’- GCAGCTCTAGGAGCATGTGG -3’ |
|  | Acsl4 | F 5’- CCTGAGGGGCTTGAAATTCAC -3’  R 5’- GTTGGTCTACTTGGAGGAACG -3’ |
|  | Il6 | F 5’- CTGCAAGAGACTTCCATCCAG -3’  R 5’- AGTGGTATAGACAGGTCTGTTGG -3’ |
|  | Alox5 | F 5’- GGGCTGTAGCGAGAAGCATC -3’  R 5’- CACGGTGACATCGTAGGAGT -3’ |
|  | Gpx4 | F 5’- TGTGCATCCCGCGATGATT -3’  R 5’- CCCTGTACTTATCCAGGCAGA -3’ |
|  | Gapdh | F 5’-AGGTCGGTGTGAACGGATTTG-3’  R 5’-GGGGTCGTTGATGGCAACA-3’ |


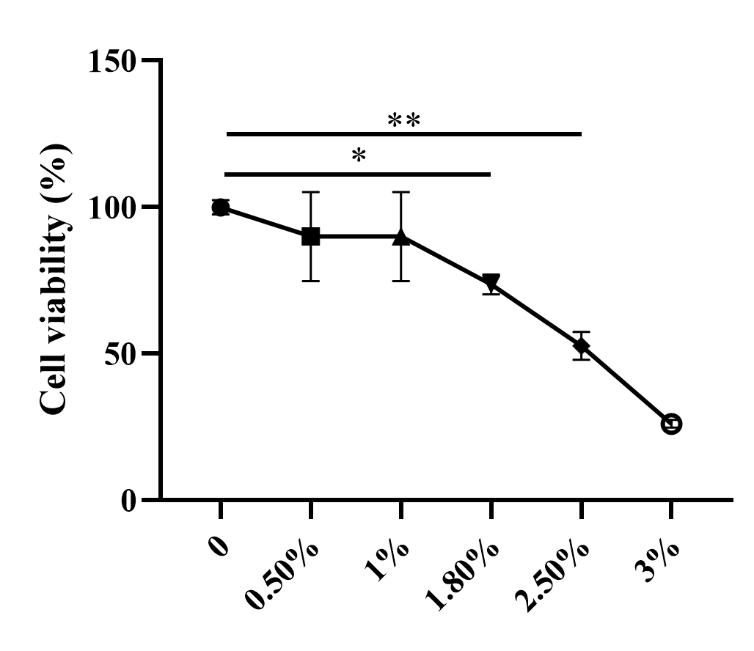


Figure S1. The cell viability of NCM460 cells in different DSS concentrations.


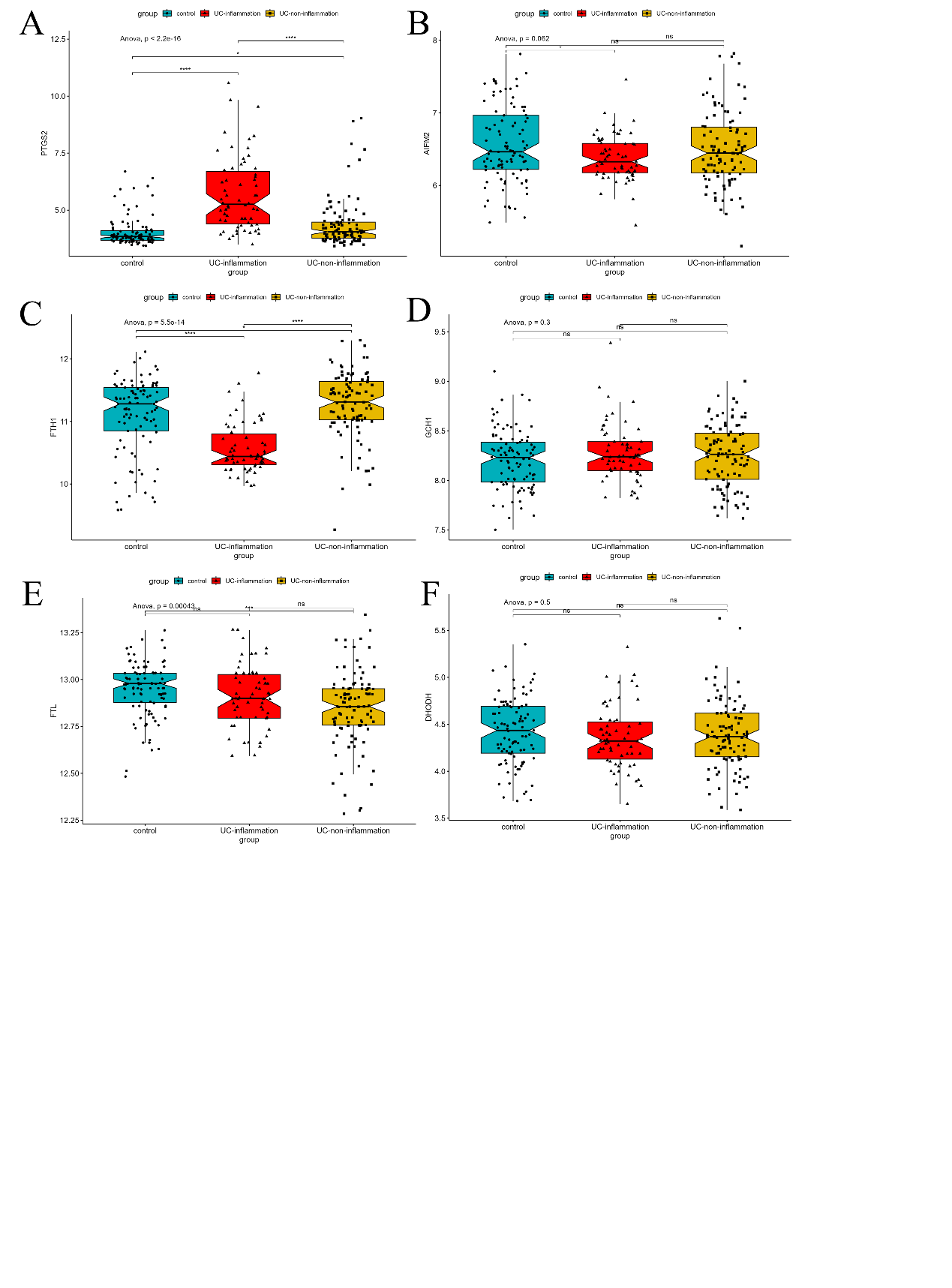


Figure S2. The expression of ferroptosis related genes. (A) PTGS2; (B) AIFM2; (C) FTH1; (D) GCH1; (E) FTL; (F) DHODH.
